# Integrative transcriptome- and DNA methylation analysis of brain tissue from the temporal pole in suicide decedents and their controls

**DOI:** 10.1038/s41380-023-02311-9

**Published:** 2023-11-08

**Authors:** Qiong Sha, Zhen Fu, Martha L. Escobar Galvis, Zach Madaj, Mark D. Underwood, Jennifer A. Steiner, Andrew Dwork, Norman Simpson, Hanga Galfalvy, Gorazd Rozoklija, Eric D. Achtyes, J. John Mann, Lena Brundin

**Affiliations:** 1 Department of Neurodegenerative Science, Van Andel Institute, Grand Rapids, MI USA; 2 Bioinformatics & Biostatistics Core, Van Andel Institute, Grand Rapids, MI USA; 3 Core Technologies and Services, Van Andel Institute, Grand Rapids, MI USA; 4 Department of Psychiatry, Columbia University College of Physicians and Surgeons, New York, NY USA; 5 Division of Molecular Imaging and Neuropathology, New York State Psychiatric Institute, New York, NY USA; 6 Department of Pathology and Cell Biology, Columbia University College of Physicians and Surgeons, New York, NY USA; 7 Macedonian Academy of Sciences and Arts, Skopje, Macedonia; 8 Division of Mental Health Data Science, New York State Psychiatric Institute, New York, NY USA; 9 Pine Rest Christian Mental Health Services, Grand Rapids, MI USA; 10 Department of Psychiatry, Western Michigan University Homer Stryker M.D. School of Medicine, Kalamazoo, MI USA

**Keywords:** Neuroscience, Depression

## Abstract

Suicide rates have increased steadily world-wide over the past two decades, constituting a serious public health crisis that creates a significant burden to affected families and the society as a whole. Suicidal behavior involves a multi-factorial etiology, including psychological, social and biological factors. Since the molecular neural mechanisms of suicide remain vastly uncharacterized, we examined transcriptional- and methylation profiles of postmortem brain tissue from subjects who died from suicide as well as their neurotypical healthy controls. We analyzed temporal pole tissue from 61 subjects, largely free from antidepressant and antipsychotic medication, using RNA-sequencing and DNA-methylation profiling using an array that targets over 850,000 CpG sites. Expression of *NPAS4*, a key regulator of inflammation and neuroprotection, was significantly downregulated in the suicide decedent group. Moreover, we identified a total of 40 differentially methylated regions in the suicide decedent group, mapping to seven genes with inflammatory function. There was a significant association between *NPAS4* DNA methylation and *NPAS4* expression in the control group that was absent in the suicide decedent group, confirming its dysregulation. *NPAS4* expression was significantly associated with the expression of multiple inflammatory factors in the brain tissue. Overall, gene sets and pathways closely linked to inflammation were significantly upregulated, while specific pathways linked to neuronal development were suppressed in the suicide decedent group. Excitotoxicity as well as suppressed oligodendrocyte function were also implicated in the suicide decedents. In summary, we have identified central nervous system inflammatory mechanisms that may be active during suicidal behavior, along with oligodendrocyte dysfunction and altered glutamate neurotransmission. In these processes, NPAS4 might be a master regulator, warranting further studies to validate its role as a potential biomarker or therapeutic target in suicidality.

## Introduction

Suicide is a leading cause of death, with more than 700,000 cases registered across the globe each year [1]. Suicide tragically affects all age groups, including adolescents, pregnant and post-partum women, as well as elderly individuals. It is the most common cause of death due to non-accidents for people below the age of 35 years [2]. Mood disorders such as major depressive disorder (MDD) are commonly associated with suicide [3], with as many as 60% of those who die by suicide having had a diagnosis of MDD [4]. MDD has an elevated rate of both suicidal ideation, and nonfatal and fatal suicide attempts [5]. Suicidal behavior is thought to have a multi-factorial etiology. Some of the most robust risk factors are socio-economic circumstances, male biological sex, and childhood exposure to traumatic events [6–8]. Suicidal behavior is estimated to have a heritability of 30–50% [9, 10], yet specific risks genes remain to be identified. Recent large-scale GWAS studies suggest that suicidal behavior is linked to multiple genes with smaller effect sizes, rather than few genes with large effect [11–13].

Biologically, suicidal behavior has repeatedly been linked to increased levels of inflammatory factors, such as IL-6 and TNF-α, in peripheral blood and cerebrospinal fluid (CSF) [14–16]. Other lines of evidence that potentially can be linked to inflammation, implicate altered hypothalamic pituitary adrenal (HPA)-axis functioning as well as a dysfunctional serotonergic system in suicidal individuals [17]. However, there is sparse information about the underlying biological mechanisms within the brain from patients at a time of active suicidal behavior, due to the inherent difficulties in obtaining these samples. Pandey et al. found evidence that cytokines, inflammasomes as well as Toll-like receptors (TLR) are upregulated in postmortem brain tissue from suicide decedents compared to controls [18]. Other studies have documented altered glial activity in suicide decedents [19]. Epigenetic changes in patients who died from suicide have also been highlighted, with changes in the methylation of genes such as *NR3C1*, *SKA2* and *PSORS1C3* reported to associate with suicide [20–22].

We sought, in an unbiased fashion, to identify transcriptomic changes significantly linked to suicide in temporal pole tissue and to overlay these findings with changes in DNA methylation, followed by further pathway analyses. A genomic approach that emphasizes identifying neural mechanisms involved in suicidal behavior, can potentially further the development of prevention and treatment strategies. This study included subjects (*N* = 61) who were largely unmedicated with psychotropic medications immediately prior to death, as verified by a postmortem toxicology analysis of brain tissue. All subjects in the suicide decedent group (*N* = 29) had MDD based on a SCID interview of significant others and none of the control cases (*N* = 32) had a psychiatric disorder or died from suicide. We assayed the transcriptome in bulk tissue by RNA-sequencing and performed DNA-methylation analysis by Illumina EPIC array.

## Material and methods

### Institutional approvals and cohort characteristics

This study was approved by the Institutional Review Boards at Columbia University, New York, USA and Van Andel Institute, Grand Rapids, Michigan, USA. Sixty-one brain samples were collected postmortem from 21 females and 40 males, age ranging from 17 to 77 years of age. Twenty-nine subjects were suicide decedents, and the remaining 32 subjects were individuals who died suddenly from other causes including accidents, homicide or myocardial infarction and serve as controls. Next-of-kin gave written informed consent for the interviews and tissue donation as required by the local Institutional Review Board. Inclusion criteria: a diagnosis of Major Depressive Disorder (MDD) and death ruled as suicide, based on postmortem examinations and SCID interviews with family, as detailed below. Inclusion criteria for controls were sudden death by homicide, accident, or natural causes. Exclusion criteria: alcohol or substance use disorder, liver disease/changes with alcoholism, brain neuropathological changes, a verdict of undetermined death, mental retardation, any chronic illness affecting CNS function, resuscitation with over 19 min of hypoxia, and any damage to brain region of interest.

### Postmortem brain tissue collection

Postmortem brain tissue from the temporal pole was provided by the New York Psychiatric Institute and Columbia University Psychiatric Disorder Macedonian/NYSPI Brain Collection. Details of brain collection and storage and screening for neuropathology and drugs have been described previously [23]. Subjects who had experienced a prolonged agonal period were excluded from the study. Briefly, all brains were collected at autopsy, sliced into 2 cm coronal slices and frozen in a step-down fashion using freezing solutions before storage at −80 °C. The pathologist performing the dissection was blind to future study design, hypotheses, and analytical methods. The temporal pole, Brodmann Area 20, gray and white matter were dissected for the assays. Samples of right perirhinal gyrus (BA36) were taken from the 2 cm thick frozen coronal slice of the right cerebral hemisphere at the level of the rostral end of the amygdala. The sample was removed from the most ventral part of the gyrus through the full thickness of the cortical ribbon. This was done with a dental drill while the tissue was at −80 °C. The tissues collected from the two groups did not differ significantly for postmortem interval (PMI) or tissue quality. The median PMI for both groups was 15 h with an inter-quartile range (IQR) between 11–19 h for the suicide decedent group and 9–18 h for the control group. The median RNA integrity number (RIN) was 7 for the suicide decedents (IQR 6-8) and 6 for the controls (IQR 5-8). Median tissue integrity number (TIN) was 74 (IQR 73-74) for the suicide decedents and 73 for the controls (IQR 71-74).

### Cohort characteristics

After giving written informed consent, significant others were interviewed by an experienced clinician using the SCID-I for DSM diagnoses [24] and a battery of rating scales for clinical and developmental details including treatment history in the three months prior to death (SCID-II [25], Brown-Goodwin Lifetime History of Aggression Scale [26], and the Columbia Suicide History Form [27]). The diagnostic approach has been validated in a subset of patients with available psychiatric hospital records [28]. 29 suicide decedents were diagnosed with major depressive disorder (MDD) as their primary psychiatric diagnosis using the SCID-I at the psychological autopsy [24]. Only one of these subjects had a secondary psychiatric diagnosis, obsessive compulsive disorder (OCD). The groups did not differ significantly for sex and age with 34% of the subjects in both the suicide decedent group and the control group being women, and a mean age of 52.9 and standard deviation (SD) of 15.7 in the suicide decedent group and 48.9 (SD = 17.9) in the control group.

### Toxicology screening

Using bodily fluids obtained at autopsy and brain tissue, every case and control was assayed for the presence of drugs including major classes of psychotropic medications. Details are as follows: Alcohol (g/100 ml) in blood and urine were determined by the Widmark method or by headspace gas chromatography with flame ionization detection (HS-GC-FID). For brain tissue toxicology, frozen fragments of cerebellum were analyzed for 192 of the most commonly prescribed or abused medications/substances at the Biomarkers Laboratory Resources (Core Lab) in the Irving Institute for Clinical and Translational Research, Columbia University. Briefly, 200 micrograms cerebellar cortex is homogenized with deuterated standards in 800 microliters of water and extracted with ammonium formate/methanol and formic acid/acetonitrile. Precipitated protein is removed by centrifugation, and phospholipids, which can suppress ionization of the target molecules, are removed by solid phase extraction. The eluent is dried and reconstituted in water/methanol/acetonitrile. Compounds of interest elute between 0.6 and 12 min and are detected by positive electrospray ionization mass spectrometry (ESI-MS/MS) mass spectrometry. All target compounds are identified using the Multiple Reaction Monitoring mode with two transitions for each compound: one transition for identification and quantification, and the other for confirmation. To validate positive findings from the toxicology screen, the laboratory used quantitative targeted Ultra Performance Liquid Chromatography-mass spectrometry/mass spectrometry (UPLC-MS/MS) assays. The quantification limit of each drug standard is lower than 5 ng/ml for all compounds. Outcomes are quantified by signal/noise ratio. The results of the brain tissue toxicology analysis are shown in Supplementary Table 1. Additionally, the mean blood alcohol level for the suicide decedent group was 0.011 (SD = 0.03) and the control group was 0.023 (SD = 0.08); the mean urine alcohol level for the suicide decedent group was 0.021 (SD = 0.05) and the control group was 0.031 (SD = 0.11). The alcohol group differences were not statistically significant.

### Construction and sequencing of RNA-seq libraries

Libraries were prepared by the Van Andel Genomics Core from 500 ng of total RNA using the KAPA RNA HyperPrep Kit (Kapa Biosystems, Wilmington, MA USA). Ribosomal RNA material was reduced using the QIAseq FastSelect –rRNA HMR Kit to remove cytoplasmic and mitochondrial rRNAs and QIAseq FastSelect –5S/16S/23S Kit to remove pan bacterial rRNAs (Qiagen, Germantown, MD, USA). RNA was sheared to 300-400 bp. Prior to PCR amplification, cDNA fragments were ligated to adapters for Illumina TruSeq UD Indexed adapters (Illumina Inc, San Diego CA, USA). Quality and quantity of the finished libraries were assessed using a combination of Agilent DNA High Sensitivity chip (Agilent Technologies, Inc.), QuantiFluor® dsDNA System (Promega Corp., Madison, WI, USA), and Kapa Illumina Library Quantification qPCR assays (Kapa Biosystems). Barcodes that are unique to each sample (from each patient) were added to each DNA fragment; and resulting DNA libraries were pooled (combined) in the sequencing run. The sequencing was performed on an Illumina NovaSeq6000 targeting depth of 100 M per sample with 100 bp, paired-end configuration. Base calling was done by Illumina Real Time Analysis and output of NextSeq Control Software was demultiplexed and converted to Fastq format with Illumina Bcl2fastq v1.9.0.

### Computational analyses

Sample sizes were set a-priori and informed via a power analysis using published methylation data from a similar study to gather plausible variance and effect-size ranges [29]. Based on the estimates, we calculated >90% power to detect relative differences of +/−15% in beta values between suicide decedents and controls, while maintaining a < 5% false discovery rate (assuming a true null proportion ~0.1%).

### Transcriptome data analysis

Raw RNA-sequencing reads were first trimmed using program Trim Galore (v0.6) to remove low quality bases and adapter sequences [30]. Next, trimmed reads were aligned to human reference genome (hg38) using STAR (2.7.3a) [31]. Count tables of all samples were then imported into edgeR (v2.34.1) [32]. Normalization was carried out using “calcNormFactors” function in edgeR which utilizes TMM method (trimmed mean of M-values) to estimate scale factors between samples [32]. Genes that had less than 0.5 count per million (cpm) normalized counts and presented in less than 20 (30% of all the samples) samples were excluded from the downstream analysis. Transcript integrity numbers (TIN) were used to assess transcript quality. TIN was calculated by RSeQC (4.4.0) [33]. Differential gene expression was evaluated by general linear modeling with a likelihood ratio test by including sex and age as covariates in the model. Gene Set Enrichment Analysis was performed using clusterProfiler (3.0.4) [34] and msigdbr (7.5.1) [35] in R (4.2.1). Gene sets included were hallmark, C2, C5, C6, C7 and KEGG. *P*-values were adjusted by applying the Benjamini-Hochberg method to control false discovery rate (FDR). FDR < 0.05 was deemed to be statistically significant.

### NPAS4 correlation analysis

Correlation analysis between DNA methylation and gene expression for the *NPAS4* gene was conducted by non-parametric robust linear regression using R (v4.2.1) (https://cran.r-project.org/) via the MASS package (7.3-58.1) [36]. The average gene-level beta-values were rank-ordered and used as the dependent variable; rank-order gene expression was modeled as the explanatory variable. Differential correlation analysis between *NPAS4* and other genes, as well as gene sets between the suicide decedent and control group were conducted using the function “ddcorAll” from the DGCA package with default parameter settings (1.0.2) [37]. Correlation analysis between *NPAS4* and other genes, regardless of group, was conducted via the function “generateCorrelations” from the correlationAnalyzeR package with default parameter settings (1.0.0) [38]. *P*-values were adjusted by applying the Benjamini-Hochberg method to maintain a 5% false discovery rate.

### DNA methylation profiling and data analysis

DNA methylation was assessed using Infinium MethylationEPIC Kit from Illumina at The New York Genome Center [39]. Differential DNA methylation analysis including sex and age as covariates was conducted by SeSAMe (1.14.2) [40] in R (4.2.1). SeSAMe was employed for two analyses: differential methylation locus (DML) and differential methylation region (DMR). DML utilizes mixed linear models to identify DMLs among groups from DNA methylation values (β values; sex and age were adjusted in our models). Based on the DML results, DMRs combines neighboring CpGs displaying consistent methylation patterns to form differentially methylated regions. Enrichment analysis was conducted with methylGSA-ORA (apply over-representation analysis) (1.14.0) [41]. Hypermethylation and hypomethylation were defined by higher and lower beta values in the suicide decedent group compared to the healthy controls.

### Deconvolution analysis

Deconvolution analysis was conducted using SCDC (0.0.0.9000) [42] and MuSiC (0.2.0) [43], which are two distinct deconvolution methods tailored for bulk RNA-seq. These methods leverage cell-type specific gene expressions derived from multiple single-cell RNA-seq reference datasets. Single nucleus reference was used from a previously published dataset [44] and sequencing data were downloaded from the Allen Brain Bank [45]. Group comparison of cell-type population differences was conducted using mixed-effects models via the Template Model Builder with beta family, logit link, and unstructured covariance matrix [46]; *P*-values were multiple testing adjusted using Tukey via the *emmeans* R package (1.8.1-1) [47]. The difference in the ratio of inhibitory and excitatory neurons between groups was estimated using robust linear mixed-effects models (3.1.3) [48]; *P*-values were multiple testing adjusted via Tukey within the *emmeans* package (1.8.1-1) [47] in R (4.2.1).

## Results

### Differential gene expression analysis (DE)

**Fig. 1:**
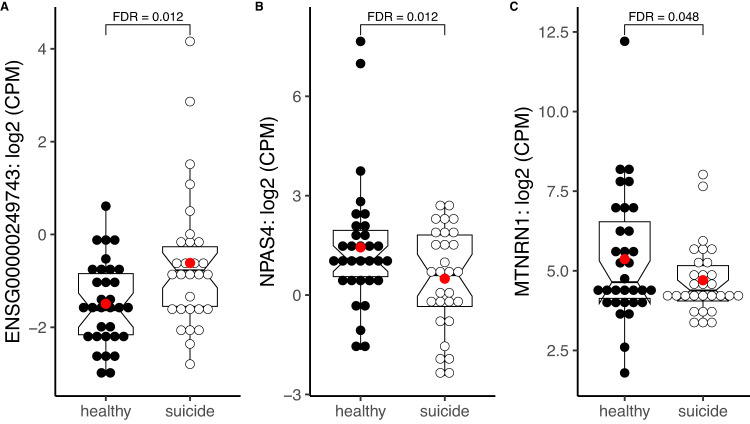
Differentially expressed genes in temporal cortical brain tissue from suicidal decedents versus nonsuicide, nonpsychiatric controls. *N* for healthy = 32; *N* for suicide decedent = 29. Lines in the notched boxplots showing 25, 75 percentiles and medians. **A** Normalized counts per million (CPM) of *ENSG00000249743*; **B** CPM of *NPAS4*; **C** CPM of *MT-NRN1*. *P*-values were adjusted by applying the Benjamini-Hochberg method to control false discovery rate (FDR).

We performed RNA-sequencing on postmortem temporal cortex brain tissue from 61 subjects (29 from the suicide cohort and 32 from neurotypical donors). Next, we conducted DE expression analysis and identified three genes to be differentially expressed after FDR correction (Supplementary Table 2). Two genes were downregulated in the suicide decedent group: *NPAS4* (log_2_FC = −2.66, FDR = 0.012) and *MTRNR1* (log_2_FC = −2.16, FDR = 0.048) (Fig. 1B, C). One gene transcript, *ENSG00000249743* (Fig. 1A), is a long non-coding RNA (lncRNA), expressed by gene located on chromosome 5, and was found to be upregulated in suicide decedents.

### Gene set enrichment analysis (GSEA)

To determine if any gene set or pathway was enriched in the suicide decedent group, we performed enrichment analysis on the selected database from GSEA, including the following gene sets: Hallmark gene sets (H); Curated gene sets (C2-CGP); GO-molecular process (C5-BP); GO-cellular component (C5-CC); GO-molecular function (C5-MF); oncogenic signature gene set (C6); immunologic signature gene set (C7).

**Fig. 2:**
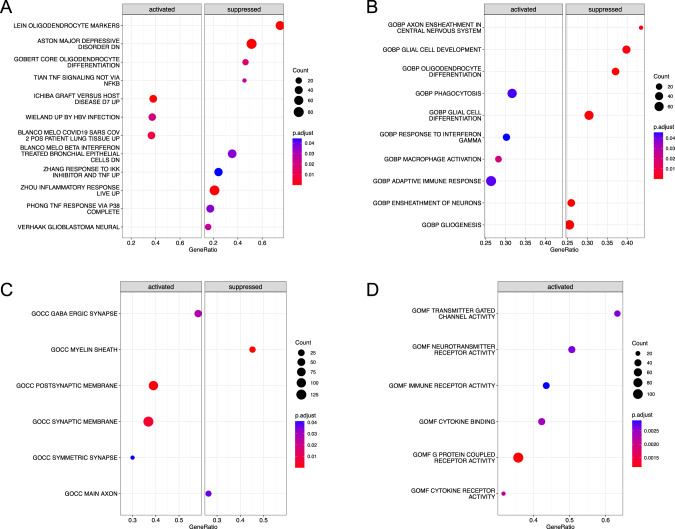
Gene set enrichment analysis results for selected gene sets. **A** Curated gene sets, subcollections: Chemical and genetic perturbations (C2-CGP); **B** Ontology gene sets, subset: Biological Process ontology (C5-BP); **C** Ontology gene sets, subset: Cellular Component ontology (C5-CC); **D** Ontology gene sets, subset: Molecular Function ontology (C5-MF).

We identified several gene sets and pathways to be enriched in the suicide decedent group that were related to inflammation and neuronal development. For instance, in H, 4/5 of the enriched gene sets were related to inflammation; in C2, 17/114 enriched gene sets were related to inflammation and infection, and 13/114 were related to immune system. In the C2-CGP database (3384 gene sets), 114 were significant, 17 of which were primarily related to inflammation and infection (13 additional datasets related to immune systems) (Fig. 2A). In the C5-BP database (7658 gene sets), 44 were significant, nine of which were primarily related to inflammation and infection (Fig. 2B). Interestingly, from the Ontology gene set (C5), Protein Demethylation Activity was upregulated in the suicide decedent group C5-BP (normalized enrichment score (NES) = 1.96, FDR = 0.013) (Fig. 2B) and C5-MF (NES = 1.92, FDR = 0.046) (Fig. 2D). 21/1009 gene sets were enriched in the C5-CC database, representative ones were shown in Fig. 2C. Representative enriched pathways from GSEA database are shown in Fig. 2. Complete significant enriched gene sets are listed in Supplementary Table 3.

**Fig. 3:**
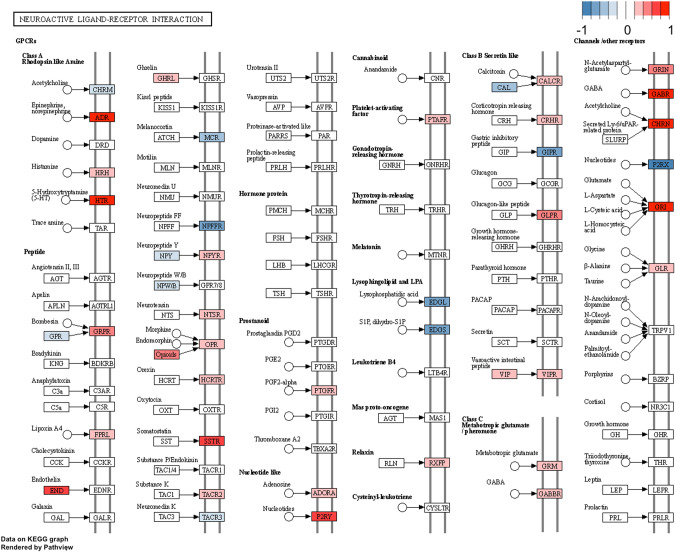
KEGG pathway analysis showing neuroactive ligand receptor interaction was significantly different between the suicide decedent and control group. Red: suicide decedent group had higher gene expression. Blue: suicide decedent group had lower gene expression.

Next, we conducted a pathway analysis. From the KEGG pathway database, nine pathways were found to be enriched in the suicide decedent group, and of these, three were associated with inflammation and infection. For example, the neuroactive ligand receptor interaction differed between the suicide decedents and the healthy control group. In addition, several neuro-relative receptors encoding genes such as *GABA*, *CHRN*, *ADR* and *HTR* were upregulated in the suicide decedent group (Fig. 3).

### NPAS4 correlation analyses

We were interested in finding out if the expression of *NAPS4* was correlated with the expression of any other genes. To determine this, we conducted a genome-wide correlation analysis between *NPAS4* and the other 17,304 unique genes in our data set and found 75 genes to be significantly correlated with *NPAS4* (FDR < 0.05). Among these 75 genes, 19 were inflammation- or immune function-related including *FOSB* (correlation coefficient = 0.766, FDR = 0.004) and *JUNB* (correlation coefficient = 0.611, FDR = 0.013). Genes that are significantly correlated with *NPAS4* regardless of group are listed in Supplementary Table 4.

**Fig. 4:**
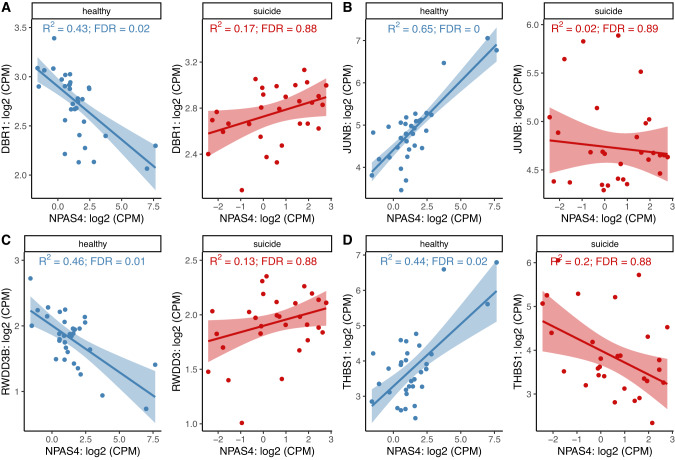
*NPAS4* related differential gene-pair correlations between the suicide decedent group and controls. **A** *NPAS4* and *DBR1* FDR = 0.026, **B** *NPAS4* and *JUNB* FDR = 0.018, **C** *NPAS4* and *RWDD3B* FDR = 0.029, and **D** *NPAS4* and *THBS1* FDR = 0.01.

We further investigated if the correlations between *NPAS4* and other genes or gene sets were different between the suicide decedent and the control group. For this purpose, we conducted a differential correlation analysis. We detected five unique genes that had a significantly different correlation pattern with *NPAS4* between the two groups (FDR < 0.05). Four out of these were inflammation- and immune system related (Fig. 4A), namely *DBR1*, *JUNB*, *RWDD3B* and *THBS1*. The other significant gene is *SP2-DT* (FDR = 0.01), which was negatively correlated with *NPAS4* in the healthy control group but positively correlated with *NPAS4* in the suicide decedent group. We also found that the correlation between the *NPAS4* expression and certain sets of genes was significantly different between the suicide decedent and the control group. For example, in the gene oncology biological process (GO-BP) gene set, 63 gene sets showed a significantly different association with *NPAS4* between the two groups; 20 of these gene sets are associated with inflammation (Supplementary Fig. 1).

### DNA methylation analysis

**Fig. 5:**
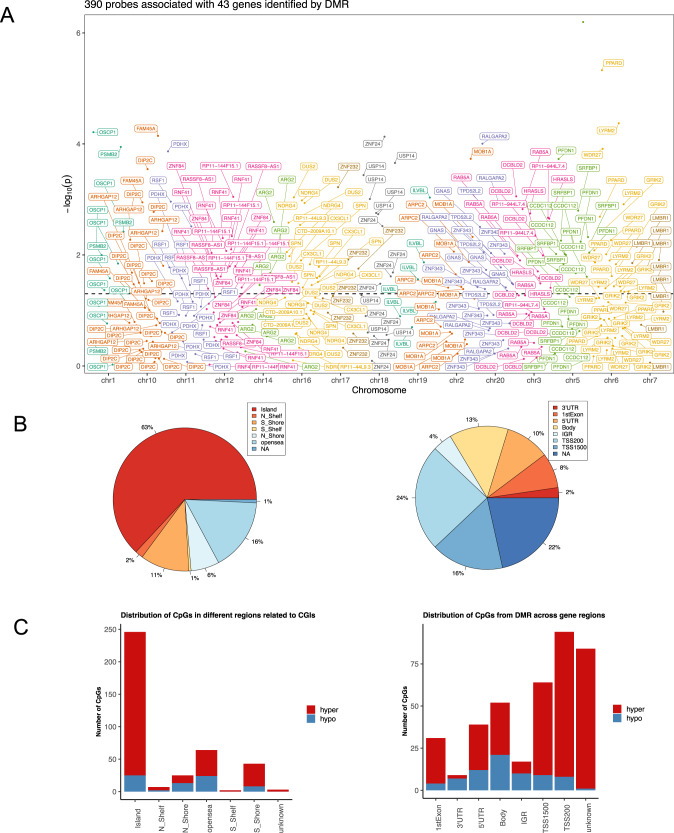
DNA methylation analysis. **A** Manhattan plot of DMRs on chromosomes. DMR analysis identified 40 regions that were significantly differentially methylated between suicide and control group (FDR < 0.05). 390 probes were within these regions. This figure displays the distribution of the 43 genes that are overlapped with these 390 probes on the chromosomes. Dashed line showed significance cut-off at FDR = 0.05. There are no genes that overlapped with DMRs located on chromosome 4, 8, 9, 13, 15, 21, 22 or 23. **B** CpGs distribution in different regions related to CpG islands (CGIs) (right); CpGs distribution in different gene regions (left). N_shore, S_shore refers to the 2,000 base sequences, directly up- and downstream of CpG islands are called the northern and southern shore respectively. N_shelf, S_shelf refers to the 2,000 base sequences directly adjacent to the shores are called the northern and southern shelves respectively. Opensea refers to the DNA methylation sites outside the CpG island regions. TSS1500 refers to 200–1,500 bases upstream of the transcriptional start site (TSS). TSS200 means 0–200 bases upstream of TSS. 5′UTR stands for the 5′ untranslated region located between the TSS and the ATG start site. 1stExon is short for the first exon of the gene. Body is the region between ATG start site and stop codon. 3′UTR is short for 3′ untranslated region that is between stop codon and poly-A tail. Intergenic region (IGR) is a stretch of DNA sequences located between genes. **C** The number of hyper- and hypomethylated CpGs from DMR (*N* = 390). Hypermethylation and hypomethylation is defined if suicide decedent group had higher or lower beta values than the control group.

A total of 670,298 probes remained for DNA methylation analysis after standard quality control measures were undertaken. We found that over 6% of these probes (40,421) were differentially methylated (DML) in the suicide decedent group after adjusting for sex and age. There are 40 DMR after applying a chromosome-wide multiple comparison test that included 390 probes. These 40 regions were mapped to 43 genes (FDR adjusted *P*-value < 0.05). Descriptions of the significant genes and probes identified by DMR are listed in Supplementary Table 5. A display of the mapped genes on each chromosome is shown in Fig. 5A. Out of these 43 genes, seven genes were related to inflammation and immune response, *ARPC2*, *CX3CL1*, *PSMB2*, *RNF41*, *RSF1*, *SPN* and *USP14*; and an additional four genes were associated with neural development and signaling including *GRIK2*, *NDRG4*, *PRARD* and *ZNF24*.

We further investigated the location of differentially methylated probes identified in DMR that have been corrected for multiple comparisons. Among all the samples, regardless of suicide status, 46% of the CpG sites were on the island, 15% were on the shore and 15% were in the open sea area (Fig. 5B left). Feature distribution shows that the majority of the CpG sites were at promoter regions such as TSS200 (24%) and TSS1500 (16%) (Fig. 5B right). Across the whole genome, 318 out of 390 probes (81.5%) were hypermethylated in the suicide decedent group. For CpG sites on the CpG islands, the majority (63%) of the methylation sites are within the island region and 90% of the probes in this region were hypermethylated in the suicide decedent group (Fig. 5C left). An examination of gene location revealed that increased hypermethylation was most prominent in the promoter regions such as TSS200, TSS1500 and 1st exon, as 91.5%, 85.9% and 87.1% respectively of the probes in these regions were hypermethylated (Fig. 5C right).

### DNA methylation enrichment analysis

Using enrichment analysis, we found the following pathways were enriched in the suicide decedent group: endoplasmic reticulum associated degradation (ERAD) pathway, lipid modification, cell surface interactions at the vascular wall, endoplasmic reticulum (ER)-Golgi intermediate compartment, lysosome, ER to Golgi anterograde transport, transport to Golgi and subsequent modification, asparagine *N*-linked glycosylation, Golgi to ER retrograde transport, intra-Golgi and retrograde Golgi to ER transport, heat shock protein binding, phenol containing compound metabolic process, cell adhesion molecules, and purine metabolism (Supplementary Fig. 2).

### Gene expression and DNA methylation correlation analysis

Combining the genes within the significant DMRs and enriched gene sets, we found 54 differentially methylated genes (DM). After a comparison with the top 50 DE genes from the RNA-sequencing analysis (Supplementary Table 2), there was no overlap. However, three pairs of paralogs were found including *ARHGAP11A* (DE analysis) and *ARHGAP12* (DM analysis), *TP53INP2* (DE analysis) and *TPD52L2* (DM analysis), *NR4A1* (DE analysis) and *NR4A2* (DM analysis). A correlation analysis between gene expression and DNA methylation for the DE gene *NPAS4* showed that the healthy controls had a positive correlation (*P* = 0.042, non-parametric robust linear regression). However, this correlation was not present in the suicide decedent group (*P* = 0.188). Moreover, the correlation between *NPAS4* expression and its methylation was different between the healthy and suicide decedent group (*P* = 0.022) (Supplementary Fig. 3). Finally, enrichment analysis from RNA-sequencing also identified gene sets that are related to DNA methylation including Protein Demethylase Pathway and Protein Demethylation (GO-MF, NES = 1.92, FDR = 0.049, GO-BP, NES = 1.90, FDR = 0.049, respectively).

### Deconvolution analysis

Deconvolution analysis showed that most of the cells from the brain tissue were astrocytes, excitatory neurons, inhibitory neurons, oligodendrocytes (OLs) and OLs precursor cells (OPCs). A representative cell type distribution figure is shown in Supplementary Fig. 4 (from the MTG reference brain region, using SCDC).

**Fig. 6:**
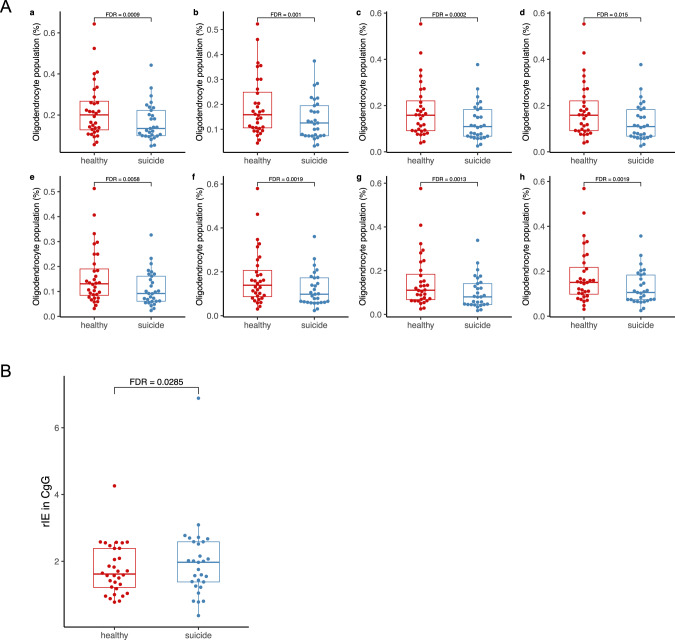
Deconvolution analysis. **A** Oligodendrocyte populations in control *vs* suicide decedent group from 8 referenced brain regions (population data generated by MuSiC, glmmTMB for group comparisons, adjusting for sex and age). a: V1C: primary visual cortex; b: S1ul: upper limb of primary somatosensory cortex; c: S1lm: lower limb of primary somatosensory cortex; d: CgG: anterior cingulate gyrus; e: MTG: middle temporal gyrus; f: M1ul: upper limb of middle temporal gyrus; g: M1lm: lower limb of middle temporal gyrus; h: A1C: primary auditory cortex. Group comparison of cell-type population differences was conducted using mixed-effects models via the Template Model Builder with beta family, logit link, and unstructured covariance matrix. **B** The ratio of inhibitory neuron counts and excitatory neuron counts (rIE) in suicide and healthy groups referenced against CgG regions using deconvoluted data generated by SCDC using robust mixed-effect linear regression, adjusted for sex, age, and multiple comparisons (multiple comparisons were conducted among the 8 brain regions as listed in Fig. 6A).

Compared with controls, the suicide decedent group had fewer OLs (FDR < 0.05) (Fig. 6A). Moreover, when using the CgG region as a reference, the suicide decedents had higher inhibitory/excitatory neuron ratio (rIE=total number of inhibitory neurons/total number of excitatory neurons) compared with the control group (FDR = 0.029) (Fig. 6B), and statistical trend for high rIE in the suicide decedent group in both the VIC and A1C regions (FDR = 0.06 and 0.07 respectively; SCDC deconvolution, robust linear mixed-effects models adjusted for age and sex). Using MuSiC deconvoluted data, we found similar trends, although none of these regions reached a statistically significant level (Supplementary Fig. 5).

## Discussion

We analyzed brain tissue from the temporal pole of suicide decedents and group matched controls by subject level integration of both transcriptome profiles and DNA methylation profiles. Design strengths of this study stemmed from use of brain samples from subjects in both the suicide group and the control group who were not on psychotropic medication at the time of death, and the groups were sex and age matched. Furthermore, all the subjects who died from suicide were diagnosed with MDD, based on postmortem SCID interviews with family members and significant others. This relatively homogenous set of participants is of importance in order to reduce variability in the transcriptome and methylation datasets due to medication exposure or diagnostic heterogeneity.

In the transcriptome analysis, we identified two genes encoding proteins with known function that were differentially expressed between the two groups after FDR correction. *NPAS4*, which was down regulated in the suicide decedent group, encodes a calcium dependent transcription factor, which regulates genes involved in the homeostasis of excitatory-inhibitory balance and neuroprotection [49]. NPAS4 has been shown to play an essential role in neuronal circuit development, neural plasticity and differentiation, as well as in inflammation and mitochondrial function [50–52]. NPAS4 also regulates brain derived neurotrophic factor (BDNF) and spatial features of neuronal inhibition, and its overexpression in cell culture induces *BDNF* expression [53]. In vivo studies in rodent models have shown that NPAS4 has a neuroprotective role in cerebral ischemia and epilepsy [54]. Other animal models have indicated that NPAS4 could have a role in fear memory, anxiety, social- and depressive-like behavior [55] and *Npas4* knockout mice display social anxiety, hyperactivity, memory and learning deficits [49, 56, 57]. NPAS4 deficiency in mice results in an increase in activated microglia and astrocytes as well as increased protein levels of IL-6 and TNF-α after stroke [54]. There are only sparse reports on *NPAS4’*s expression in humans thus far. Interestingly, recently Sosnowski et al. found that *NPAS4* is downregulated in dorsolateral prefrontal cortex tissue from subjects who died from opioid overdose [58].

We next performed correlation analyses, showing that *NPAS* expression was significantly associated with inflammation-related factors such as *JUNB*, *FOSB*, *DBR1*, *RWDD3* [59–62]. The observed correlations between *NPAS4* and *JUN* and *FOSB*, respectively, were at least partially to be expected, since all three genes are activity-dependent transcription factors and have been shown to increase early in studies of neuronal activation. Moreover, FOS and JUN together form the AP-1 complex which is known to regulate dynamic cellular responses, including immune activation [61, 62]. Interestingly, the associations between *NPAS4* and three other genes *JUNB*, *DBR1* and *RWDD3* were significantly different in the suicide decedent group compared to in controls, possibly indicating a deficient regulatory effect of NPAS4 on the expression of these genes in suicide decedents. Our results not only show that *NPAS4* expression was reduced in temporal cortex from suicide decedents, but also a positive correlation between *NPAS4* expression and methylation in healthy controls that was not present in the suicide decedent group.

The second gene that was significantly differentially expressed at the whole-genome level in suicide decedents was *MTRNR1*, a mitochondrially encoded 12S rRNA gene. *MTRNR1* has previously been linked to auditory neuropathy spectrum disorder and deafness and is associated with mitochondrial dysfunction [63]. In line with this finding, a potential role for mitochondrial metabolism in suicide by violent means has been suggested in data derived from a previous large transcriptome analysis [64].

Many factors can differ between cohorts examined in depression/suicide studies (e.g., the use of psychotropic medicines, type of tissue studied, demographic factors), making direct comparisons between transcriptomic studies difficult. In our current study, the suicide decedents were also diagnosed with MDD as confirmed by a careful postmortem SCID interview with family members. Interestingly, among our top ranked DE genes, we identified three genes previously found to be differentially expressed in tissues from patients with MDD [65]. Specifically, *CTNNA3* (cell-cell adhesion, ranked 47th), *HSPA1A* (heat shock protein, ranked 22nd) and *PSORS1C1* (confers susceptibility to psoriasis and systemic sclerosis, ranked 88th) in our top 100 DE gene list were also associated with MDD as described in a genome-wide meta-analysis involving 807,553 individuals [65]. However, the relatively low number of overlapping genes in our study with depression-related genes might suggest that the biological mechanisms involved in active suicidal behavior are mainly different from those in MDD without suicidal behavior. Recently, using a co-expression network analysis, another group identified 309 genes that were associated with suicidal behavior and a set of module genes including heat shock protein family A member 1B (*HSA1B*), *HSA1A*, GNAS complex locus (*GNAS*), estrogen receptor 1 (*ESR1*), as well as five pathways including estrogen signaling pathway [66]. Interestingly, *HSPA1B* and *HSPA1A* were ranked 60 and 53 by the lowest *P*-value in our DE gene list, while *GNAS* was a differentially methylated gene in our suicide decedent group. Moreover, estrogen signaling was also close to significant in our transcriptome enrichment analysis (KEGG pathway analysis, FDR = 0.053).

As our next step, we performed an enrichment analysis, which showed that several gene sets and pathways related to inflammation, infection, and neuronal development were both differentially expressed and methylated in the suicide decedents. Overall, the suicide decedent group showed an activation of gene sets related to inflammation and excitotoxic mechanisms, accompanied by a suppression of gene sets related to maturation of OLs and myelination. The deconvolution analysis indicated an increased ratio of inhibitory neurons against excitatory neurons in the suicide decedent group and further an imbalance in several neurotransmitters, as illustrated in Supplementary Fig. 1. The balance of inhibitory and excitatory neurotransmitter levels plays a significant role in several psychiatric disorders including MDD [67, 68]. Interestingly, the concentration of these neurotransmitters might not only be associated with the numbers of their interneurons but also with their functions [69, 70].

Moreover, utilizing deconvolution analysis, we found that the suicide decedent group had fewer OLs than the healthy control group in all reference brain regions. In line with this, Aston and colleagues have previously reported abnormalities of oligodendroglia in the temporal pole from patients with MDD by transcriptome analysis [71]. Our enrichment analysis demonstrated that the suicide decedent group exhibited suppression of a gene set that was also found to be down regulated in Aston’s study in MDD (Fig. 2A). Our enrichment analysis identified 10 gene sets and pathways that relate to OLs development and myelination process. Genes encoding the key myelination-related proteins Myelin Basic Protein (MBP) and Myelin Associated Oligodendrocyte Basic protein (MOBP) ranked 11th and 25th in our DE gene list. Consistent with our findings, previous transcriptome analyses of suicide postmortem frontal cortex tissue found a lower expression of genes involved in OLs differentiation [19] and a reduction of OPCs in subjects who died from a violent suicide compared to a non-violent suicide [64]. We also found the gene *ZNF24* to be significantly hypermethylated in the suicide decedent group. ZNF24 (also known as ZFP24, ZFP191) is a transcriptional regulator, and phosphorylation of ZNF24 controls the developmental process of oligodendrocyte progenitor cells to pre-myelinating OLs [72] and as such it is required by OLs during myelination [73]. We detected that almost all the *ZNF24* CpGs were located in the promotor region and hypermethylated in the suicide decedent group, consistent with suppression of gene expression [74].

Previous studies have shown that OLs and their precursors are vulnerable to inflammation, oxidative stress and elevated glutamate levels [75], which explains why these cells are affected in multiple neuropathological entities, including Alzheimer’s disease, spinal cord injury, Parkinson’s disease, and ischemia, as well as during hypoxia [76, 77]. OL deficits have also been previously reported in schizophrenia and bipolar disorder [78, 79] and direct evidence of altered oxidative stress markers has been detected in OLs from MDD suicide postmortem brain tissues [80]. The coincidence of gene expression patterns between upregulation of immune response and down regulation of maturation of OLs in suicide decedents in our study might suggest a causal relationship between the two. Indeed, OPCs were shown to be cytotoxic targets of neuroinflammation in a study of the demyelinating disease multiple sclerosis [81]. Also, there is experimental evidence that OLs block their own differentiation in response to inflammation by activating toll-like receptor-3 (TLR3) [82]. Deconvolution analysis heavily relies on the reference datasets used. While we utilized a widely cited single-cell sequencing dataset from the human brain [44], it is worth noting that the brain regions in this dataset do not precisely align with Brodmann Area 20, as we were unable to find a single-cell dataset specific only to this region. However, in the regions utilized for deconvolution, there is a partial overlap with Brodmann Area 20, and we were able to identify a consistent pattern of lower oligodendrocyte cell populations in the suicide decedent group compared to the control group. To further validate our findings and address the regional specificity, conducting future single-cell RNA sequencing specifically from Brodmann Area 20 would be valuable.

Another recent integrative DNA methylation (via Infinium human 450 BeadChip) and gene expression analysis (via Illumina HumanHT-12 V4 Expression BeadChip) on postmortem brain tissue from male suicide decedents identified 622 differentially expressed genes in the suicide decedent group compared with controls [83]. Among them, 70 genes had concordant methylation and expression changes including genes relevant to psychiatric disorders such as *ADCY9*, *CRH*, *NFATC4*. None of these genes were differentially expressed or methylated in our study. That study differs from our current one in that more than half of the subjects in the previous study had a history of substance abuse, and many, including the controls, had a variety of psychiatric disorders [83]. In contrast, the suicide decedents in our study were largely free from psychotrophic medication, as confirmed with postmortem toxicology, and had a confirmed diagnosis of MDD. The region of analysis, the prefrontal cortex, was also different from our current study as we utilized tissue from the temporal pole, another region proposed to be involved in suicidal behavior [84, 85]. We have previously conducted a pilot study analyzing prefrontal cortical tissue and found evidence of both hypermethylation and a focus of findings in inflammation and neurotrophic pathways [86, 87].

### Limitations of this study

Our DNA methylation analysis used Illumina EPIC array platform which is designed to cover 30% of the human methylome [88]. Thus, inferences made from these methylation profiles require cautious interpretation. Moreover, our DNA methylation analysis only detects the CpG sites while it has been shown that non-CpG methylation can also play a role in both neurons and glial cells [89], especially later in life [90]. Furthermore, our sample sizes are relatively small for the Illumina Epic 850k array, so it is likely that we have missed additional differentially methylated regions [91]. Another limitation is the dissection process of the brain tissue, which can lead to slight variation in the anatomical region used for analysis. Since the dissected brain gyri fold in three dimensions, it is impossible to exclude all white matter while still taking the full thickness (~ 3 mm) of the cortical ribbon. However, it is important to note that any random variation during dissection should not impact the groups differently, as pathologists were blind to future study design and analytical approaches. Despite the limitations of this study, it is important to note that the data presented here offer important indications of distinct molecular signatures in well-characterized suicidal individuals, without any major influence of psychotrophic medication, and that they can serve as the basis for designing future targeted studies.

## Conclusions

Overall, our study suggests a network of mechanisms involved in suicidal behavior, centering on upregulated inflammatory pathways and the suppression of oligodendrocyte-related genes. It is plausible to propose that in the brain, NPAS4 may serve as a master transcriptional regulator that modulates neural and neuronal circuit development while maintaining mitochondrial and immune function. *NPAS4* downregulation could lead to excitatory and inhibitory imbalance, impaired neural development, increased oxidative stress and neuroinflammation. Our results confirm the involvement of inflammatory pathways in active suicidal behavior and suggest that NPAS4-associated mechanisms might serve as novel targets in the development of therapies for suicide prevention. Further, this work also supports that the role of OLs should be further evaluated in suicidal behavior.

### Supplementary information

Supplementary Figures

Supplementary Table 1.

Supplementary Table 2.

Supplementary Table 3.

Supplementary Table 4.

Supplementary Table 5.

## Data Availability

The codes used in this project can be found at https://github.com/psychesha21/RNAseq_Analysis and https://github.com/psychesha21/DNA-methylation. Sequencing data for RNA and DNA methylation can be accessed by GEO accession number: SuperSeries GSE243488.
