# Supplementary material for: Integrative transcriptome- and DNA methylation analysis of brain tissue from the temporal pole in suicide decedents and their controls
Source: Mol Psychiatry. 2023 Nov 8;29(1):134–45. doi: 10.1038/s41380-023-02311-9 (PMC11078738; doi:10.1038/s41380-023-02311-9)
Supplement: Supplementary file 1 — Supplementary Figures [file 41380_2023_2311_MOESM1_ESM.docx]

**Supplementary Figure 1**

Supp Fig. 1 Gene sets related with inflammation and immune functions in the gene ontology biological process (GO-BP) database that had significantly different correlations with *NPAS4* between suicide and control group. Multiple comparison was adjusted by Bonferroni method.

**Supplementary Figure 2**

Supp Fig. 2 Significantly enriched gene sets and pathways in the suicide group by methylGSA-ORA analysis. A: Enriched gene sets and pathways including all CpG regions; B: Enriched gene sets and pathways in the P1 promoter region (including TSS200, TSS1500); C: Enriched gene sets and pathways in the gene body region (including body and 1^st^ exon).

**Supplementary Figure 3**

Supp. Fig. 3 Non-parametric robust linear regression between DNA methylation estimate and gene expression log2 fold change associated with gene *NPAS4*.

**Supplementary Figure 4**

Supp. Fig. 4 Cell type population distribution by SCDC using MTG brain region as reference. Left: Healthy control; Right: Suicide group. OPC: oligodendrocyte precursor cell. Excitatory neurons including: "IT", "L6b","L6.CT", "L5.6.IT.Car3", "L4.IT”. Inhibitory neurons including: “VIP", "LAMP5", "SST", “PAVLB”. Cell Type Nomenclature see Allen Brian Bank.

**Supplementary Figure 5**


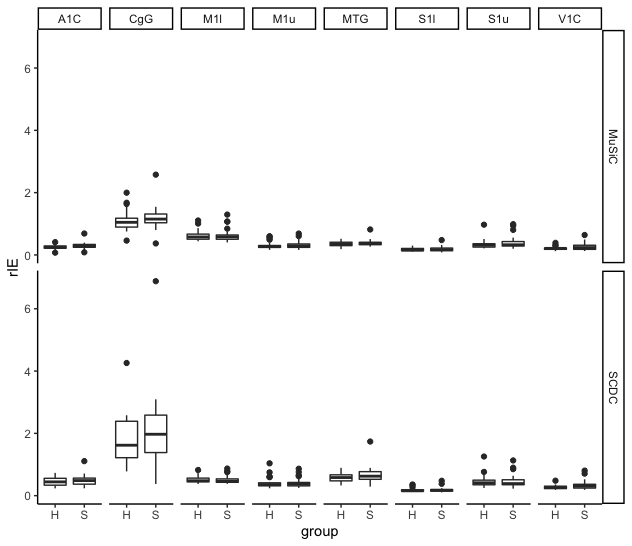


Supp Fig. 5 Inhibitory/excitatory neuron ratios (rIE) from all brain regions using both SCDC and MuSiC. H: Healthy control; S: Suicide group.
