# Supplementary material for: Integrative transcriptome- and DNA methylation analysis of brain tissue from the temporal pole in suicide decedents and their controls
Source: Mol Psychiatry. 2023 Nov 8;29(1):134–45. doi: 10.1038/s41380-023-02311-9 (PMC11078738; doi:10.1038/s41380-023-02311-9)
Supplement: Supplementary file 3 — Supplementary Table 2. [file 41380_2023_2311_MOESM3_ESM.docx]

**Supplementary Table 2.** Top 50 differentially expressed genes in suicide decedents versus healthy controls.

| **Gene Symbol** | **logFC** | ***P*-value** | **FDR** |
| --- | --- | --- | --- |
| **ENSG00000249743** | **2.02252995** | **5.70E-07** | **0.01178457*** |
| **NPAS4** | **-2.6593917** | **1.09E-06** | **0.01178457*** |
| **MT-NRN1** | **-2.1588577** | **6.69E-06** | **0.0481251*** |
| ARHGAP11A | -0.3897861 | 3.72E-05 | 0.20111026 |
| MTURN | -0.5351917 | 5.40E-05 | 0.22138145 |
| ENSG00000234665 | 1.33137173 | 7.37E-05 | 0.22138145 |
| TP53INP2 | -0.9195281 | 8.77E-05 | 0.22138145 |
| PAIP2B | -0.9366908 | 9.26E-05 | 0.22138145 |
| CHRNA3 | 1.51992829 | 9.54E-05 | 0.22138145 |
| LINC00632 | -0.4813426 | 1.03E-04 | 0.22138145 |
| MBP | -1.170859 | 1.21E-04 | 0.23727247 |
| ADGRF5P1 | 0.79537375 | 1.45E-04 | 0.26166221 |
| PAQR6 | -0.832445 | 1.85E-04 | 0.26727087 |
| ENSG00000266844 | -1.2183174 | 1.89E-04 | 0.26727087 |
| TMEFF1 | -0.5460529 | 1.89E-04 | 0.26727087 |
| KIF5B | -0.3716768 | 1.98E-04 | 0.26727087 |
| ANKRD40 | -0.4985456 | 2.30E-04 | 0.29240298 |
| LPAR1 | -0.8667611 | 2.53E-04 | 0.30305153 |
| BCAS1 | -0.929213 | 3.28E-04 | 0.37253662 |
| MAP4 | -0.3922162 | 3.61E-04 | 0.38676066 |
| ENSG00000257657 | -0.4281779 | 3.76E-04 | 0.38676066 |
| PKP4 | -0.4655691 | 4.11E-04 | 0.39554007 |
| ABCA12 | 0.90285486 | 4.57E-04 | 0.39554007 |
| TRAK2 | -0.3070641 | 4.73E-04 | 0.39554007 |
| HIPK2 | -0.7134174 | 4.91E-04 | 0.39554007 |
| MOBP | -1.0708146 | 4.93E-04 | 0.39554007 |
| ACTA2 | 0.81988201 | 4.95E-04 | 0.39554007 |
| SEPTIN4 | -0.6832968 | 5.71E-04 | 0.42810257 |
| HLA-DPB2 | 1.2379153 | 5.75E-04 | 0.42810257 |
| SYNDIG1L | 0.84932914 | 6.00E-04 | 0.43160167 |
| LINC01137 | -0.589618 | 6.48E-04 | 0.45111356 |
| SH3PXD2A | -0.4394082 | 7.44E-04 | 0.46898829 |
| ENSG00000230313 | 1.14769582 | 7.74E-04 | 0.46898829 |
| LOC728485 | 0.30411281 | 7.75E-04 | 0.46898829 |
| ENSG00000270021 | -0.6517382 | 7.77E-04 | 0.46898829 |
| KIF1C | -0.6665726 | 7.82E-04 | 0.46898829 |
| ZEB2 | -0.379355 | 8.93E-04 | 0.49955026 |
| PTP4A2 | -0.3641617 | 8.95E-04 | 0.49955026 |
| ENSG00000274492 | 0.3956469 | 9.02E-04 | 0.49955026 |
| GAB2 | -0.5353966 | 9.49E-04 | 0.502 |
| MTND1P23 | -1.2967202 | 9.53E-04 | 0.502 |
| ENSG00000226816 | 0.50733038 | 9.99E-04 | 0.5048363 |
| SSTR4 | 0.53767434 | 0.00100518 | 0.5048363 |
| DYNC1LI2 | -0.3155962 | 0.00112799 | 0.53247213 |
| WEE2-AS1 | 0.45303169 | 0.00115958 | 0.53247213 |
| FGF1 | -0.5974961 | 0.00117883 | 0.53247213 |
| CTNNA3 | -0.88044 | 0.00119547 | 0.53247213 |
| CDK6 | -0.4114611 | 0.00126882 | 0.53247213 |
| NR4A1 | -0.7429928 | 0.00128442 | 0.53247213 |
| ENSG00000184258 | -0.6125057 | 0.00128668 | 0.53247213 |
