# Supplementary material for: Integrative transcriptome- and DNA methylation analysis of brain tissue from the temporal pole in suicide decedents and their controls
Source: Mol Psychiatry. 2023 Nov 8;29(1):134–45. doi: 10.1038/s41380-023-02311-9 (PMC11078738; doi:10.1038/s41380-023-02311-9)
Supplement: Supplementary file 6 — Supplementary Table 5. [file 41380_2023_2311_MOESM6_ESM.docx]

**Supplementary Table 5**. Significant genes and probes within the DMR.

| **Gene name** | **Chromosome** | **Probe ID** | **Estimate** | **P value** | **Description** |
| --- | --- | --- | --- | --- | --- |
| ARG2 | 4 | cg00122215 | -0.0014965 | 0.54562879 | Arginase 2 catalyzes the hydrolysis of arginine to ornithine and urea. |
|  |  | cg04822973 | 0.00162975 | 0.22370317 |  |
|  |  | cg06830503 | -0.005584 | 0.18076416 |  |
|  |  | cg09360748 | 0.00049491 | 0.09441889 |  |
|  |  | cg10043376 | 0.00059095 | 0.0348736 |  |
|  |  | cg13051728 | 0.00028884 | 0.22017024 |  |
|  |  | cg13633669 | 0.00031261 | 0.29231481 |  |
|  |  | cg16682385 | 0.00092422 | 0.02114862 |  |
|  |  | cg18458993 | 0.00122735 | 0.00254356 |  |
|  |  | cg20477070 | 0.00580658 | 0.00962985 |  |
|  |  | cg23702868 | 0.00107861 | 0.01027843 |  |
| ARHGAP12 | 10 | cg01859118 | 0.0005518 | 0.16686455 | ARHGAP12 encodes a member of a large family of proteins that activate Rho-type guanosine triphosphate (GTP) metabolizing enzymes. |
|  |  | cg03454028 | 0.00131455 | 0.00532259 |  |
|  |  | cg09110292 | -0.0056332 | 0.3448187 |  |
|  |  | cg11493139 | 0.00134371 | 0.03163657 |  |
|  |  | cg13593941 | 0.00075563 | 0.06116013 |  |
|  |  | cg18675610 | -0.0086409 | 0.365779 |  |
|  |  | cg20095920 | 0.00157814 | 0.18520966 |  |
|  |  | cg25648639 | 0.00499433 | 0.00373428 |  |
|  |  | cg26784012 | -0.0142208 | 0.0156768 |  |
| ARPC2 | 2 | cg01978704 | 0.00051056 | 0.19569497 | Actin Related Protein 2/3 Complex Subunit 2 helps to promote actin polymerization in the nucleus. |
|  |  | cg05351940 | 0.00069586 | 0.04652137 |  |
|  |  | cg09247346 | 0.00104055 | 0.03886437 |  |
|  |  | cg11691181 | 0.0006287 | 0.2614935 |  |
|  |  | cg11838463 | 0.00148519 | 0.00871491 |  |
|  |  | cg13027571 | 0.00090143 | 0.02229315 |  |
|  |  | cg13240332 | 0.00084423 | 0.60063996 |  |
|  |  | cg14561349 | 0.00094348 | 0.00614035 |  |
|  |  | cg16672685 | 0.00077286 | 0.04112057 |  |
| CCDC112 | 5 | cg09241467 | 0.0086301 | 0.00961281 | Coiled-Coil Domain Containing 112 |
|  |  | cg10297710 | 0.00130564 | 0.00999776 |  |
|  |  | cg11745506 | 0.00082016 | 0.07168395 |  |
|  |  | cg12882476 | 0.00082576 | 0.04931903 |  |
|  |  | cg13013671 | 0.00042251 | 0.21707211 |  |
|  |  | cg17231906 | 0.00907654 | 0.00685264 |  |
|  |  | cg19688118 | 0.00960049 | 0.02128258 |  |
|  |  | cg19920737 | 0.00066321 | 0.67078858 |  |
|  |  | cg20948757 | 0.00154615 | 0.2347703 |  |
|  |  | cg25267732 | 0.00578952 | 0.02471936 |  |
| CTD-2009A10.1 | 16 | cg01961256 | -0.0030859 | 0.20133879 | lincRNA |
|  |  | cg03979726 | -0.0056099 | 0.01523048 |  |
|  |  | cg13440073 | -0.0119531 | 0.0736721 |  |
| CX3CL1 | 16 | cg04452432 | 0.01978921 | 0.01635669 | This gene belongs to the CX3C subgroup of chemokines. |
|  |  | cg15195412 | 0.01501241 | 0.01474204 |  |
|  |  | cg20427865 | 0.01880419 | 0.10474774 |  |
|  |  | cg20816757 | 0.01310134 | 0.05888773 |  |
|  |  | cg22549797 | 0.01589546 | 0.04245955 |  |
|  |  | cg26644853 | 0.01952026 | 0.02593005 |  |
| DCBLD2 | 3 | cg02464093 | 0.00109158 | 0.00088041 | Discoidin, CUB and LCCL Domain Containing 2 is  involved in negative regulation of cell growth and wound healing. |
|  |  | cg04416750 | 0.00068243 | 0.0535577 |  |
|  |  | cg05950791 | -0.0009174 | 0.74522375 |  |
|  |  | cg07435592 | -0.0064305 | 0.23926737 |  |
|  |  | cg07906351 | 0.00091531 | 0.12190476 |  |
|  |  | cg09615786 | 0.00126609 | 0.00377971 |  |
|  |  | cg14396960 | 0.00082274 | 0.70956807 |  |
|  |  | cg16180217 | 0.00215747 | 0.02621343 |  |
|  |  | cg17567125 | 0.0057599 | 0.01022171 |  |
|  |  | cg27352765 | 0.00134463 | 0.03913017 |  |
|  |  | cg27410952 | -0.001202 | 0.27764609 |  |
| DIP2C | 10 | cg04630908 | -0.0047115 | 0.00156731 | DIP2C encodes a member of the disco-interacting protein homolog 2 family. |
|  |  | cg05483131 | -0.0014415 | 0.02090095 |  |
|  |  | cg05484691 | -0.0037923 | 0.04268179 |  |
|  |  | cg07629149 | -0.0005803 | 0.73346509 |  |
|  |  | cg09805925 | -0.0027541 | 0.04529544 |  |
|  |  | cg12227954 | 0.00359809 | 0.52399201 |  |
|  |  | cg12537302 | -0.0005314 | 0.55998354 |  |
|  |  | cg13351028 | -0.0008849 | 0.4901729 |  |
|  |  | cg13387972 | 0.01437798 | 0.03245386 |  |
|  |  | cg14497145 | 0.00406305 | 0.35553536 |  |
|  |  | cg15551781 | 0.01505378 | 0.00028422 |  |
|  |  | cg15864612 | -0.0042109 | 0.06458118 |  |
|  |  | cg18170385 | 0.00684161 | 0.02965315 |  |
|  |  | cg18721155 | -0.0039657 | 0.00167783 |  |
|  |  | cg19082416 | 0.00039244 | 0.8436526 |  |
|  |  | cg21712619 | -0.0044234 | 0.169005 |  |
|  |  | cg23844018 | -0.0009397 | 0.04645682 |  |
|  |  | cg24718710 | 0.00566321 | 0.24297075 |  |
|  |  | cg27197835 | -0.0007092 | 0.74316187 |  |
|  |  | cg27311272 | -0.0028776 | 0.25296259 |  |
| DUS2 | 16 | cg03079559 | 0.00085775 | 0.00261976 | This gene encodes a cytoplasmic protein that catalyzes the conversion of uridine residues to dihydrouridine in the D-loop of tRNA. DUS2 has been implicated in pulmonary carcinogenesis. |
|  |  | cg04528069 | 0.00107176 | 0.03053702 |  |
|  |  | cg04960941 | 0.00105695 | 0.00091594 |  |
|  |  | cg05567709 | 0.00098238 | 0.04677279 |  |
|  |  | cg06140118 | -0.0005319 | 0.26449139 |  |
|  |  | cg06703304 | 0.00105053 | 0.18132576 |  |
|  |  | cg11289251 | 0.00105393 | 0.00115856 |  |
|  |  | cg13665184 | 0.00085735 | 0.0738959 |  |
|  |  | cg17982504 | 0.00051154 | 0.04567344 |  |
| FAM45A | 10 | cg06579345 | 0.00071709 | 0.16111075 | Also known as DENND10, FAM45A enables guanyl-nucleotide exchange factor activity and small GTPase binding activity. |
|  |  | cg12847800 | 0.00171696 | 0.00112857 |  |
|  |  | cg13527016 | 0.00373095 | 0.0896256 |  |
|  |  | cg20227259 | 0.00081052 | 0.14087736 |  |
|  |  | cg20943499 | 0.00224286 | 7.16E-05 |  |
| GNAS | 20 | cg03264550 | -0.0271965 | 0.00313041 | Guanine Nucleotide Binding Protein (G Protein), Alpha. |
|  |  | cg07909402 | -0.0366422 | 0.01861262 |  |
|  |  | cg14104369 | -0.0248557 | 0.03165994 |  |
|  |  | cg25130962 | -0.0275948 | 0.03223072 |  |
|  |  | cg25983380 | -0.0252056 | 0.03379074 |  |
| GRIK2 | 6 | cg05942459 | 0.00714314 | 0.14091531 | This gene product belongs to the kainate family of glutamate receptors, which are composed of four subunits and function as ligand-activated ion channels. |
|  |  | cg06247406 | 0.01068545 | 0.18506631 |  |
|  |  | cg10591607 | 0.01687827 | 0.00337146 |  |
|  |  | cg13080565 | 0.00575691 | 0.30953715 |  |
|  |  | cg15081698 | 0.0079496 | 0.1413476 |  |
|  |  | cg16009558 | 0.0095223 | 0.05508437 |  |
|  |  | cg17881572 | 0.00774915 | 0.4363422 |  |
|  |  | cg18193094 | 0.0100786 | 0.03675164 |  |
|  |  | cg21635870 | 0.00534132 | 0.01715992 |  |
|  |  | cg22541254 | 0.01166682 | 0.03693931 |  |
|  |  | cg24301620 | 0.00827506 | 0.28803492 |  |
|  |  | cg24753760 | 0.00878357 | 0.21094423 |  |
|  |  | cg26316946 | 0.01189787 | 0.0722443 |  |
|  |  | cg27074174 | 0.00210312 | 0.6097886 |  |
|  |  | cg27451362 | 0.02044352 | 0.05695828 |  |
| HRASLS | 3 | cg08659357 | 0.0010606 | 0.00179009 | Also known as PLAAT1, the HRASLS subfamily enables acyltransferase activity and possesses phospholipase activity. |
|  |  | cg16419066 | 0.00109485 | 0.03255965 |  |
|  |  | cg20436262 | 0.00048568 | 0.09406832 |  |
|  |  | cg24710320 | 0.00100043 | 0.00221816 |  |
| ILVBL | 19 | cg06718723 | 0.00655405 | 0.12726583 | Mutations in IlvB Acetolactate Synthase Like are associated with aspirin-exacerbated respiratory disease. |
|  |  | cg09395833 | 0.00635247 | 0.04483147 |  |
|  |  | cg15575671 | 0.00419363 | 0.19988544 |  |
|  |  | cg20262129 | 0.00530492 | 0.02749262 |  |
|  |  | cg21059382 | 0.00092652 | 0.01362239 |  |
|  |  | cg23023178 | 0.00179527 | 0.00092631 |  |
|  |  | cg25667171 | 0.00529641 | 0.07003278 |  |
| LMBR1 | 7 | cg02565050 | 0.00233136 | 0.01399265 | LMBR1 encodes Limb Development Membrane Protein 1. An intron of *LMBR1* serves as the cis-acting regulatory module for the sonic hedgehog (*SHH*) gene. |
|  |  | cg03936250 | 0.00669504 | 0.0070619 |  |
|  |  | cg07681084 | 0.00041192 | 0.18129688 |  |
|  |  | cg10063233 | 0.00055384 | 0.0191226 |  |
|  |  | cg13324717 | 8.96E-05 | 0.87179143 |  |
|  |  | cg14664464 | 0.00094649 | 0.03491989 |  |
|  |  | cg16133845 | 0.00124844 | 0.0123346 |  |
|  |  | cg17536848 | 0.01215808 | 0.10519444 |  |
|  |  | cg25359978 | 0.00095289 | 0.05170491 |  |
| LYRM2 | 6 | cg02725437 | 0.0058951 | 0.06059862 | Diseases associated with LYRM2 include [Multiple Mitochondrial Dysfunctions Syndrome 2 With Hyperglycinemia](https://www.malacards.org/card/multiple_mitochondrial_dysfunctions_syndrome_2_with_hyperglycinemia) |
|  |  | cg03200341 | 0.00206691 | 0.33613866 |  |
|  |  | cg04843946 | 0.00258169 | 0.03741013 |  |
|  |  | cg10392378 | 0.00765992 | 0.14825213 |  |
|  |  | cg11240327 | 0.0012849 | 0.03976248 |  |
|  |  | cg12849807 | 0.00027698 | 0.5200756 |  |
|  |  | cg20011833 | 0.00721996 | 0.01470643 |  |
|  |  | cg21264329 | 0.00101672 | 0.54081574 |  |
|  |  | cg21621759 | 0.00042093 | 0.16278756 |  |
|  |  | cg21980685 | 0.00167344 | 4.23E-05 |  |
|  |  | cg25134683 | 0.00119897 | 0.00122092 |  |
|  |  | cg25430506 | 0.00112306 | 0.04623742 |  |
|  |  | cg25719236 | -0.0020614 | 0.20526954 |  |
|  |  | cg27642643 | 0.00366924 | 0.13697981 |  |
| MOB1A | 2 | cg00327537 | 0.00061915 | 0.09581888 | The protein encoded by MOB1A is a component of the Hippo signaling pathway, which controls organ size and tumor growth by enhancing apoptosis. |
|  |  | cg06966617 | 0.00026374 | 0.53173916 |  |
|  |  | cg08434152 | 0.00268785 | 0.36273032 |  |
|  |  | cg10626925 | 0.00052617 | 0.24453185 |  |
|  |  | cg16083546 | 0.0010272 | 0.03775286 |  |
|  |  | cg17034940 | -0.0036485 | 0.33364087 |  |
|  |  | cg19885631 | 0.00478215 | 0.40398573 |  |
|  |  | cg20340806 | 0.0016048 | 0.01342064 |  |
|  |  | cg23149560 | 0.01423482 | 0.00018538 |  |
|  |  | cg23724873 | 0.00226507 | 0.00472894 |  |
|  |  | cg24415849 | 0.00128078 | 0.06131275 |  |
| NDRG4 | 16 | cg00984694 | -0.0001996 | 0.67059262 | NDRG4 is a member of the N-myc downregulated gene family which belongs to the alpha/beta hydrolase superfamily. The protein encoded by NDRG4 is cytoplasmic and required for cell cycle progression and survival in primary astrocytes. |
|  |  | cg01466678 | 0.00207289 | 0.05876846 |  |
|  |  | cg02040433 | 0.00081614 | 0.00887746 |  |
|  |  | cg02950651 | 0.0017265 | 0.00144782 |  |
|  |  | cg04797985 | 0.00019032 | 0.79222477 |  |
|  |  | cg06650542 | -0.0046169 | 0.25674434 |  |
|  |  | cg08791131 | 0.00152677 | 0.00104055 |  |
|  |  | cg11306587 | 0.00258912 | 0.05365807 |  |
|  |  | cg13031432 | 0.00074744 | 0.11553679 |  |
|  |  | cg14242059 | 0.00050549 | 0.13802842 |  |
|  |  | cg17650822 | 0.00070529 | 0.03491725 |  |
| OSCP1 | 1 | cg00578252 | 0.00222351 | 6.14E-05 | OSCP1 enables transmembrane transporter activity and is involved in xenobiotic detoxification. |
|  |  | cg06367472 | 0.00152562 | 0.00609682 |  |
|  |  | cg08878450 | 0.00384885 | 0.08252064 |  |
|  |  | cg10035922 | 0.0055579 | 0.04304852 |  |
|  |  | cg11798976 | -0.0049535 | 0.17154502 |  |
|  |  | cg12630409 | 0.00185601 | 0.02640604 |  |
|  |  | cg14912727 | -0.0001117 | 0.94307047 |  |
|  |  | cg15725228 | 0.0022482 | 0.00140853 |  |
|  |  | cg16583330 | 0.00185605 | 0.01428956 |  |
|  |  | cg22397854 | 0.00353504 | 0.06989956 |  |
| PDHX | 11 | cg06937548 | 0.00165256 | 0.00013632 | The pyruvate dehydrogenase (PDH) complex is located in the mitochondrial matrix and catalyzes the conversion of pyruvate to acetyl coenzyme A. |
|  |  | cg08318506 | -0.0032003 | 0.24859753 |  |
|  |  | cg09194750 | 0.00017446 | 0.65244737 |  |
|  |  | cg11058730 | 0.0005474 | 0.17348879 |  |
|  |  | cg11622362 | 0.00077247 | 0.06861681 |  |
|  |  | cg14686008 | 0.00074887 | 0.01221277 |  |
|  |  | cg15745106 | -0.0023527 | 0.35941438 |  |
|  |  | cg15988239 | 0.00163093 | 0.0785376 |  |
|  |  | cg17147794 | -0.000377 | 0.84640714 |  |
|  |  | cg18508148 | 0.00067702 | 0.1745221 |  |
|  |  | cg20541753 | 0.00152314 | 0.0030645 |  |
|  |  | cg20869710 | 0.00511636 | 0.06961344 |  |
|  |  | cg22717608 | -0.0018751 | 0.13681624 |  |
|  |  | cg24088639 | 0.00120328 | 0.08521545 |  |
|  |  | cg25931580 | 0.00035631 | 0.33486768 |  |
| PFDN1 | 5 | cg01139526 | -0.0016314 | 0.59812186 | PFDN1 encodes a member of the prefoldin beta subunit family. The encoded protein is one of six subunits of prefoldin, a molecular chaperone complex that binds and stabilizes newly synthesized polypeptides. |
|  |  | cg02707576 | 0.00181727 | 0.01873785 |  |
|  |  | cg04956413 | 0.00924807 | 0.00018683 |  |
|  |  | cg07697078 | 0.00504658 | 0.19464358 |  |
|  |  | cg11924368 | 0.00369374 | 0.14291922 |  |
|  |  | cg16577540 | 0.00347779 | 0.00646493 |  |
|  |  | cg21445723 | 0.00504152 | 0.00576354 |  |
|  |  | cg22037779 | 0.00721904 | 0.14991418 |  |
|  |  | cg25132078 | -0.0011895 | 0.771317 |  |
|  |  | cg25409734 | 0.00663991 | 0.00476796 |  |
|  |  | cg25550546 | 0.00335962 | 0.00905241 |  |
| PPARD | 6 | cg00065598 | -0.0015994 | 0.67114073 | PPARD encodes a member of the peroxisome proliferator-activated receptor (PPAR) family. |
|  |  | cg00657095 | 0.00734229 | 0.0082692 |  |
|  |  | cg02943769 | 0.00071752 | 0.28791765 |  |
|  |  | cg05528533 | 0.0015115 | 0.0020474 |  |
|  |  | cg06610850 | 0.00164509 | 4.70E-06 |  |
|  |  | cg08893449 | 0.00924804 | 0.08431922 |  |
|  |  | cg15729095 | 0.00407833 | 0.01262725 |  |
|  |  | cg17499041 | 0.00683054 | 0.02412403 |  |
|  |  | cg18353713 | 0.00277998 | 0.13566733 |  |
| PSMB2 | 1 | cg00284249 | 0.00032753 | 0.35317541 | PSMB2 encodes Proteasome 20S Subunit Beta 2. Diseases associated with PSMB2 include Cystic Fibrosis. |
|  |  | cg09070503 | 0.00065794 | 0.01005035 |  |
|  |  | cg17948627 | 0.00728229 | 0.00011344 |  |
|  |  | cg18323922 | 0.00129375 | 0.00380611 |  |
| RAB5A | 3 | cg03678754 | 0.00134384 | 0.01029701 | Protein RAB-5A enables GDP binding activity, GTP binding activity, and GTPase activity. |
|  |  | cg04701661 | 0.00078049 | 0.01254517 |  |
|  |  | cg05483252 | -0.006573 | 0.45316271 |  |
|  |  | cg05722872 | 0.00079764 | 0.00149168 |  |
|  |  | cg07057042 | 0.00202925 | 0.05348758 |  |
|  |  | cg07231102 | 0.00032797 | 0.11523716 |  |
|  |  | cg12909010 | -0.0027974 | 0.38385176 |  |
|  |  | cg15571960 | 0.00131452 | 0.01977952 |  |
|  |  | cg17138393 | 0.00025543 | 0.45213075 |  |
|  |  | cg21977467 | 0.00149257 | 0.10869293 |  |
| RALGAPA2 | 20 | cg06525062 | 0.00108856 | 9.90E-05 | RALGAPA2 encodes Ral GTPase activating protein catalytic subunit alpha 2. Located in cytosol and plasma membrane. |
|  |  | cg09281539 | -0.0057523 | 0.22834124 |  |
|  |  | cg10723075 | -0.0027123 | 0.20931263 |  |
|  |  | cg13698996 | 0.00039343 | 0.42767256 |  |
|  |  | cg16420636 | 0.00084909 | 0.00128131 |  |
|  |  | cg16871527 | 0.00496109 | 0.01674823 |  |
|  |  | cg19808285 | 0.00085299 | 0.04435219 |  |
| RASSF8-AS1 | 12 | cg00353340 | 0.00327865 | 0.09462639 | RASSF8 antisense RNA 1 is a non-coding RNA Gene. |
|  |  | cg01521154 | 0.00994737 | 0.04051407 |  |
|  |  | cg02279650 | 0.00085759 | 0.07560883 |  |
|  |  | cg04936446 | 0.00104102 | 0.00377346 |  |
|  |  | cg05152561 | 0.00148554 | 0.10867074 |  |
|  |  | cg05459733 | 0.00349095 | 0.05742692 |  |
|  |  | cg07469792 | 0.00122184 | 0.0268914 |  |
|  |  | cg11513088 | 0.00047004 | 0.33611694 |  |
|  |  | cg13417331 | 0.00191674 | 0.2951849 |  |
|  |  | cg13826089 | 0.00489036 | 0.30076982 |  |
|  |  | cg13957428 | 0.00072248 | 0.01707988 |  |
|  |  | cg15849440 | 0.00692886 | 0.07951091 |  |
|  |  | cg19754190 | 0.00127358 | 0.02496461 |  |
|  |  | cg22946876 | -0.0032315 | 0.16159976 |  |
| RNA5SP298 | 10 | cg08653328 | 0.0101634 | 0.18646586 | RNA, 5S Ribosomal Pseudogene 298 is located at 10p15.3. |
|  |  | cg12326440 | 0.00422375 | 0.25257851 |  |
|  |  | cg15905865 | 0.00458424 | 0.098558 |  |
|  |  | cg19760211 | 0.00237918 | 0.721131 |  |
|  |  | cg21105876 | 0.00164497 | 0.73561384 |  |
| RNF41 | 12 | cg01307693 | 0.00333833 | 0.01908207 | RNF41 encodes an E3 ubiquitin ligase (Ring Finger Protein 41). The encoded protein plays a role in type 1 cytokine receptor signaling by controlling the balance between JAK2-associated cytokine receptor degradation and ectodomain shedding. |
|  |  | cg02339297 | -0.0002192 | 0.9445171 |  |
|  |  | cg02498626 | 0.0005694 | 0.19556123 |  |
|  |  | cg05435853 | 0.00233619 | 0.3024934 |  |
|  |  | cg05976119 | 0.00097043 | 0.02388453 |  |
|  |  | cg08761248 | 0.00049662 | 0.14229934 |  |
|  |  | cg11938453 | 0.00137588 | 0.00497162 |  |
|  |  | cg12026015 | 0.00021269 | 0.48500942 |  |
|  |  | cg13797950 | 0.00511704 | 0.00847082 |  |
|  |  | cg14825076 | 0.00136414 | 0.00607146 |  |
|  |  | cg16237595 | 0.00108248 | 0.00457912 |  |
|  |  | cg16278213 | 0.00083807 | 0.16780532 |  |
|  |  | cg18334474 | 0.00207007 | 0.04147341 |  |
|  |  | cg18956558 | 0.00793058 | 0.01588007 |  |
|  |  | cg22632663 | -0.0024791 | 0.57280712 |  |
| RP11-144F15.1 | 12 | cg04916200 | 0.00109212 | 0.00591341 | RP11-144F15.1 is an uncharacterized non-coding RNA. |
|  |  | cg05510519 | 0.00184546 | 0.3554028 |  |
|  |  | cg06109136 | 0.00062213 | 0.18298544 |  |
|  |  | cg10462751 | 0.00086067 | 0.02447158 |  |
|  |  | cg11800662 | 0.00296942 | 0.24143166 |  |
|  |  | cg12407666 | 0.00063634 | 0.38903931 |  |
|  |  | cg14881098 | 0.00160946 | 0.01603234 |  |
|  |  | cg16037021 | 0.00109006 | 0.00793377 |  |
|  |  | cg17161146 | 0.00174261 | 0.00089711 |  |
|  |  | cg17623555 | 0.00057931 | 0.19241769 |  |
|  |  | cg24053061 | 0.00187273 | 0.05573723 |  |
|  |  | cg26211838 | -0.0001818 | 0.96417517 |  |
|  |  | cg26979339 | 0.00166279 | 0.46090379 |  |
|  |  | cg27173711 | 0.00063197 | 0.03859377 |  |
| RP11-44L9.3 | 16 | cg08045431 | -0.0007995 | 0.46876642 | RP11-44L9.3 is an uncharacterized lincRNA. |
|  |  | cg08266233 | -0.0035142 | 0.00288452 |  |
|  |  | cg27135528 | -0.0060407 | 0.02438842 |  |
| RP11-944L7.4 | 3 | cg08999915 | 0.00641751 | 0.10722901 | RP11-944L7.4 is an |
|  |  | cg13348059 | 0.00293985 | 0.00451329 | uncharacterized non- |
|  |  | cg24158187 | 0.00373564 | 0.01350901 | coding RNA. |
|  |  | cg24410381 | 0.00508347 | 0.0021361 |  |
| RSF1 | 11 | cg07031665 | 0.00094301 | 0.01672348 | RSF1 encodes a nuclear protein that interacts with hepatitis B virus X protein (HBX) and facilitates transcription of hepatitis B virus genes by the HBX transcription activator. |
|  |  | cg07947315 | 0.00252716 | 0.00109685 |  |
|  |  | cg08444060 | 0.00252944 | 0.42405572 |  |
|  |  | cg11672159 | 0.0029344 | 0.12057701 |  |
|  |  | cg15105081 | 0.00118465 | 0.0262936 |  |
|  |  | cg17141500 | 0.00055671 | 0.12413067 |  |
|  |  | cg19851979 | 0.00161063 | 0.03474738 |  |
|  |  | cg20895389 | 0.00162348 | 0.12480388 |  |
|  |  | cg21215416 | -0.0005584 | 0.61016571 |  |
|  |  | cg21227845 | 0.00072557 | 0.13058791 |  |
|  |  | cg22821606 | 0.00097307 | 0.05816323 |  |
|  |  | cg25043477 | 0.00042583 | 0.18396317 |  |
| SPN | 16 | cg03549705 | -0.0172083 | 0.01051796 | SPN encodes a highly sialylated glycoprotein sialophorin (CD43) that functions in antigen-specific activation of T cells. |
|  |  | cg08480068 | -0.0314248 | 0.01254483 |  |
|  |  | cg10126903 | -0.0252867 | 0.03862122 |  |
|  |  | cg16644366 | -0.0139045 | 0.09937368 |  |
|  |  | cg26691434 | -0.0230779 | 0.03073099 |  |
|  |  | cg26769927 | -0.0306249 | 0.01639043 |  |
| SRFBP1 | 5 | cg02457282 | 0.0008365 | 0.00962259 | Serum response factor binding protein 1 enables RNA binding activity. |
|  |  | cg05531284 | -0.0050043 | 0.44453948 |  |
|  |  | cg07567973 | 0.00082633 | 0.00552146 |  |
|  |  | cg08597253 | 0.00115736 | 0.01456908 |  |
|  |  | cg12597276 | 0.00073258 | 0.05704597 |  |
|  |  | cg20403628 | 0.00192341 | 0.00159524 |  |
|  |  | cg21447975 | 0.00567259 | 0.18875782 |  |
|  |  | cg26918471 | 0.00159834 | 0.00591653 |  |
| TPD52L2 | 20 | cg03381996 | 0.00083558 | 0.00118516 | TPD52L2 encodes a member of the tumor protein D52-like family. These proteins are characterized by an N-terminal coiled-coil motif. |
|  |  | cg12626292 | 0.00147129 | 0.00926323 |  |
|  |  | cg12730202 | 0.00509351 | 0.08431416 |  |
|  |  | cg12933587 | 0.00049657 | 0.1205882 |  |
|  |  | cg21870731 | 0.00103372 | 0.02069782 |  |
| USP14 | 18 | cg00014996 | 0.0026768 | 0.05312011 | Ubiquitin Specific Peptidase 14 is involved in innate immune defense and is indispensable for synaptic development and function at neuromuscular junctions. |
|  |  | cg01000073 | 0.00166284 | 0.00036449 |  |
|  |  | cg01848352 | 0.0068299 | 0.25012742 |  |
|  |  | cg02273078 | 0.00086364 | 0.00098306 |  |
|  |  | cg10275340 | 0.0011569 | 0.00175432 |  |
|  |  | cg12559913 | 0.00405357 | 0.00184165 |  |
|  |  | cg18959473 | -0.0058025 | 0.27552439 |  |
|  |  | cg24438779 | 0.00076194 | 0.00022016 |  |
| WDR27 | 6 | cg01404182 | 0.00481766 | 0.01363221 | WDR27 encodes a protein with multiple WD repeats. |
|  |  | cg04940962 | 0.0005205 | 0.05951768 |  |
|  |  | cg07678032 | 0.00124684 | 0.00012236 |  |
|  |  | cg11379888 | 0.00061108 | 0.00973435 |  |
|  |  | cg14890866 | 0.0007335 | 0.07096231 |  |
|  |  | cg16197653 | 0.00077871 | 0.00540724 |  |
|  |  | cg17681549 | 0.00047035 | 0.48672641 |  |
|  |  | cg22307285 | 0.00038207 | 0.17429428 |  |
|  |  | cg22747398 | 0.00118486 | 0.04445954 |  |
|  |  | cg23204407 | 0.00048044 | 0.5869028 |  |
| ZNF232 | 17 | cg01362921 | -0.0067811 | 0.06055955 | Zinc finger protein 232 is located in cytosol and nucleoplasm. |
|  |  | cg04029612 | 0.00147404 | 0.10967831 |  |
|  |  | cg16850458 | 0.00073897 | 0.05455573 |  |
|  |  | cg18093563 | 0.0023413 | 0.00060207 |  |
|  |  | cg18880595 | 0.00152791 | 0.46867536 |  |
|  |  | cg20969781 | 0.00087343 | 0.01215242 |  |
|  |  | cg23762215 | 0.00061011 | 0.26093602 |  |
|  |  | cg25673211 | 0.00099601 | 0.01760759 |  |
| ZNF24 | 18 | cg01248421 | 0.00571862 | 0.12636479 | Zinc Finger Protein 24 is a transcription factor required for myelination of differentiated oligodendrocytes. |
|  |  | cg03161112 | -0.0012994 | 0.80990548 |  |
|  |  | cg05422369 | 0.00160821 | 0.28873178 |  |
|  |  | cg12725284 | 0.00268142 | 0.03298729 |  |
|  |  | cg13034837 | 0.00184311 | 0.00302607 |  |
|  |  | cg13703062 | 0.00140189 | 7.40E-05 |  |
|  |  | cg19062966 | 0.00216296 | 0.3375593 |  |
|  |  | cg21691267 | 0.00088026 | 0.02433546 |  |
|  |  | cg21778174 | 0.01092118 | 0.02066807 |  |
|  |  | cg26061898 | 0.00307034 | 0.14093728 |  |
| ZNF343 | 20 | cg01658602 | 0.00063017 | 0.03780436 | ZNF343 enables sequence-specific double-stranded DNA binding activity. |
|  |  | cg02867123 | 0.00377918 | 0.09354218 |  |
|  |  | cg07782925 | 0.00082456 | 0.00895358 |  |
|  |  | cg07889201 | 0.00859698 | 0.04114091 |  |
|  |  | cg08931782 | 0.00610917 | 0.12343188 |  |
|  |  | cg13236659 | -0.0012699 | 0.59913816 |  |
|  |  | cg16301451 | -0.0028711 | 0.27727291 |  |
|  |  | cg17374677 | 0.00076089 | 0.00715734 |  |
|  |  | cg17437852 | 0.00437064 | 0.34206678 |  |
|  |  | cg17660446 | 0.00187099 | 0.00952042 |  |
|  |  | cg17740950 | 0.00135836 | 0.03851187 |  |
|  |  | cg20546810 | -0.0024086 | 0.48488811 |  |
|  |  | cg21293622 | 0.00090391 | 0.17090153 |  |
| ZNF84 | 12 | cg03442510 | 0.00108298 | 0.01259037 | Predicted to enable DNA-binding transcription factor activity, RNA polymerase II-specific and RNA polymerase II cis-regulatory region sequence-specific DNA binding activity. |
|  |  | cg04375472 | 0.00095044 | 0.00920101 |  |
|  |  | cg09453737 | 0.0043133 | 0.08772634 |  |
|  |  | cg09552183 | 0.00050539 | 0.16511758 |  |
|  |  | cg12255362 | 0.00082317 | 0.05000612 |  |
|  |  | cg14876115 | 0.00115978 | 0.12350476 |  |
|  |  | cg20054939 | 0.00077733 | 0.27892232 |  |
|  |  | cg23839938 | 0.0008147 | 0.01150066 |  |
|  |  | cg27363310 | 0.00408859 | 0.086358 |  |

Note: Red genes are inflammation related. Blue genes are neural development related.
